# A Proteomic Study of Hemocyte Proteins from Mud Crab (*Scylla paramamosain*) Infected with White Spot Syndrome Virus or *Vibrio alginolyticus*

**DOI:** 10.3389/fimmu.2017.00468

**Published:** 2017-04-27

**Authors:** Baozhen Sun, Zhi Wang, Ziyan Wang, Xiongchao Ma, Fei Zhu

**Affiliations:** ^1^College of Animal Science and Technology, Zhejiang Agriculture and Forestry University, Hangzhou, China

**Keywords:** proteomic, hemocyte, *Scylla paramamosain*, white spot syndrome virus, *Vibrio alginolyticus*, isobaric tags for relative and absolute quantitation

## Abstract

In this study, we investigated the hemocytes’ immune response to white spot syndrome virus (WSSV) or *Vibrio alginolyticus* infection at the protein level. The differential proteomes from crab hemocytes infected with WSSV or *V. alginolyticus* were analyzed using the isobaric tags for relative and absolute quantitation approach immediately after infection. Using this approach, we identified 1,799 proteins by their by LC–MS/MS spectra and sequencing data. These included 157 upregulated proteins and 164 downregulated proteins after WSSV infection. Similarly, 243 proteins were determined to be differentially expressed during *V. alginolyticus* infection, of these, 121 were upregulated and 122 were downregulated after infection. Interestingly, among these differentially expressed proteins, 106 were up- or downregulated significantly in both WSSV and *V. alginolyticus* infection. Six genes, β-actin, myosin-9, anti-lipopolysaccharide factor isoform 4, anti-lipopolysaccharide factor 4, transketolase-like protein 2-like isoform 1, and sarcoplasmic calcium-binding protein 1 were chosen for further study. The expression of these genes all showed a trend of upregulation at 24 h post-WSSV or *V. alginolyticus* infection except for myosin-9 in response to WSSV. To confirm the protective effects of the six genes, crabs were injected with specific dsRNAs before WSSV or *V. alginolyticus* challenge. The results showed that the knockdown of these genes led to an increase in the morbidity and mortality (*P* < 0.01) rate, and a decrease in infection time in WSSV-infected crabs. During the first 84 h, knockdown of these genes also led to an increase in the morbidity rates in *V. alginolyticus* -infected crabs, and results of four genes showed a higher mortality rate than that of the control after they were knocked down. This is the first report of the proteome response in crab hemocytes during WSSV or *V. alginolyticus* infection. These findings will contribute to our understanding of the immune response to WSSV and *V. alginolyticus* infection in crabs.

## Introduction

The mud crab *Scylla paramamosain*, one of a number of commercially important crustaceans, is widely distributed along the coast line of southern China and the broader Pacific area. Little is known about the disease burden of these animals in the juvenile or adult phases of *S. paramamosain*, but susceptibility to infection has been identified during the larval stages. Several bacterial and viral pathogens have been reported to infect crabs (1). At present, bacterial infections are the main cause of crab disease and constraint in the crab farming industry in China (2, 3). In aquaculture, *Vibrio alginolyticus* is an important bacterial pathogen that would cause huge economic losses and has caused many cases of natural infection in the Chinese mitten crab (4). White spot syndrome has become the most hazardous and devastating disease in shrimp culture (5). White spot syndrome virus (WSSV) can also infect several species of crab both in the natural and experimental setting (6–8).

As we know, crustaceans including crabs only have an innate immune system to defend against invading microbes (9). The innate immune system includes phagocytosis, encapsulation, melanization, coagulation, and the release of antimicrobial peptides (AMPs) in crustaceans (9, 10). The hemocytes are the main site in crustaceans where the immune defense is mounted. Proteomics have been widely used to investigate the molecular mechanisms underlying the immune response to WSSV infection in crustaceans but there is no research available on *V. alginolyticus* infection yet (11, 12). Currently an isobaric tag-based methodology for peptide relative quantification [isobaric tags for relative and absolute quantitation (iTRAQ)] coupled to multidimensional liquid chromatography and tandem mass spectrometry enables the assessment of protein levels where four samples can be compared for their common effects (13). iTRAQ analysis has been performed to reveal the effects of *Spiroplasma eriocheiris* infection in Chinese mitten crab (*Eriocheir sinensis*) hemocytes (14). Crab hemocytes have been confirmed as the major target cells in WSSV or *V. alginolyticus* infection. The aim of this study was to investigate the hemocytes’ immune response to WSSV or *V. alginolyticus* infection at the protein level.

## Experimental Procedures

### Crab and Tissue Preparation

Healthy crabs were kept in a 50 L tank containing sea water with an air pump at room temperature in our laboratory. The crabs were bound with nylon rope quickly before injection and bleeding. WSSV was prepared and used immediately for the challenge according to a previous report (15). *V. alginolyticus* was cultured and used to challenge crabs according to the method published in a previous report (16). The hemocytes of pathogen-challenged crabs were collected for RNA isolation immediately and kept on ice to prevent RNA degradation.

### Pathogen Challenge and Mortality Count

For the pathogen challenge, healthy crabs were randomly distributed into three groups (*n* = 10 per group). All groups received injections in the third walking foot base of crabs. The control group received injections of phosphate-buffered saline (PBS) alone, the WSSV/*V. alginolyticus* groups received injections of 100 µL WSSV (10^5^ copies/mL)/*V. alginolyticus* (1.5 × 10^6^/mL) in PBS, and the dsRNA + WSSV/*V. alginolyticus* groups received injections of dsRNA (50 µg per crab) and 100 µL WSSV (10^5^ copies/mL)/*V. alginolyticus* (1.5 × 10^6^/mL). The mortality data were arranged and analyzed in Microsoft Excel 2003.

### Protein Preparation

Crab hemocytes lysates were prepared according to the previous report (14). The proteins in the supernatant were kept at −80°C for further analysis.

### iTRAQ Labeling and Strong Cation Exchange (SCX) Fractionation

For iTRAQ assays, two biological replicates of each group were prepared for the proteomics experiments. Total protein (100 µg) was taken out of each sample solution, and then the protein was digested with Trypsin Gold (Promega, Madison, WI, USA) with the ratio of protein:trypsin = 30:1 at 3°C for 16 h. After trypsin digestion, peptides were dried by vacuum centrifugation. Peptides were reconstituted in 0.5 M tetraethyl ammonium bromide and processed according to the manufacture’s protocol for 8-plex iTRAQ reagent (Applied Biosystems, CA, USA). Briefly, 1 U of iTRAQ reagent was thawed and reconstituted in 24 µL isopropanol. Samples were labeled with the iTRAQ tags. The peptides were labeled with the isobaric tags, incubated at room temperature for 2 h. The labeled peptide mixtures were then pooled and dried by vacuum centrifugation. SCX chromatography was performed with an LC-20AB HPLC Pump system (Shimadzu, Kyoto, Japan). The iTRAQ-labeled peptide mixtures were reconstituted with 4 mL buffer A [25 mM NaH_2_PO_4_ in 25% acetonitrile (ACN), pH 2.7] and loaded onto a 4.6 mm × 250 mm Ultremex SCX column containing 5 µm particles (Phenomenex, Torrance, CA, USA). The peptides were eluted at a flow rate of 1 mL/min with a gradient of buffer A for 10 min, 5–60% buffer B (25 mM NaH_2_PO_4_, 1 M KCl in 25% ACN, pH 2.7) for 27 min, 60–100% buffer B for 1 min. The system was then maintained at 100% buffer B for 1 min before equilibrating with buffer A for 10 min prior to the next injection. Elution was monitored by measuring the absorbance at 214 nm, and fractions were collected every 1 min. The eluted peptides were pooled into 20 fractions, desalted with a Strata X C18 column (Phenomenex, Torrance, CA, USA) and vacuum dried.

### LC–ESI–MS/MS Analysis Based on Triple TOF 5600

Each fraction was resuspended in buffer C [5% ACN, 0.1% formaldehyde (FA)] and centrifuged at 20,000 *g* for 10 min, the final concentration of peptide was about 0.5 µg/µL on average. Ten microliters of supernatant were loaded on an LC-20AD nanoHPLC (Shimadzu, Kyoto, Japan) by the autosampler onto a 2 cm C18 trap column (monolithic silica capillary column; Waters, USA). Then, the peptides were eluted onto a 10 cm analytical C18 column (monolithic silica capillary column; inner diameter 75 µm, Waters, USA) packed in-house. The samples were loaded at 8 µL/min for 4 min, then the 35 min gradient was run at 300 nL/min starting from 2 to 35% B (95% ACN, 0.1% FA), followed by 5 min linear gradient to 60%, then, followed by 2 min linear gradient to 80%, and maintenance at 80% B for 4 min, and finally return to 5% in 1 min. Data acquisition was performed with a TripleTOF 5600 System (AB SCIEX, Concord, ON, Canada) fitted with a Nanospray III source (AB SCIEX, Concord, ON, Canada) and a pulled quartz tip as the emitter (New Objectives, Woburn, MA, USA). Data were acquired using an ion spray voltage of 2.5 kV, curtain gas of 0.2 MPa, nebulizer gas of 0.1 MPa, and an interface heater temperature of 150. The MS was operated with an RP (reverse phase) of greater than or equal to 30,000 full width at half maximum for TOF MS scans. For isotope dilution analysis, survey scans were acquired in 250 ms and as many as 30 product ion scans were collected if exceeding a threshold of 120 counts per second (counts/s) and with a 2+ to 5+ charge state. Total cycle time was fixed to 3.3 s. Q2 transmission window was 100 Da for 100%. The signals from four-anode detectors were summed for each scan at a pulser frequency value of 11 kHz through monitoring of the 40 GHz multichannel TDC detector with four-anode channel detect ion. A sweeping collision energy setting of 35 ± 5 eV coupled with iTRAQ adjust rolling collision energy was applied to all precursor ions for collision-induced dissociation. Dynamic exclusion was set for 1/2 of peak width (15 s), and then the precursor was refreshed off the exclusion list.

### Data Analysis

Raw data files acquired from the Orbitrap were converted into MGF files using Proteome Discoverer 1.2 (PD 1.2, Thermo Fisher Scientific, Hudson, USA), 5,600 ms converter and the MGF files were searched. Protein identification was performed using the Mascot search engine (Matrix Science, London, UK, version 2.3.02). For protein identification, a mass tolerance of 0.05 Da (ppm) was permitted for intact peptide masses and 0.1 Da for fragmented ions, with allowance for one missed cleavages in the trypsin digests. Gln → pyro-Glu (N-term Q), oxidation (M), deamidated (NQ) as the potential variable modifications, and carbamidomethyl (C), iTRAQ8plex (N-term), iTRAQ8plex (K) as fixed modifications. The charge states of peptides were set to +2 and +3. Specifically, an automatic decoy database search was performed in Mascot by choosing the decoy checkbox in which a random sequence of database is generated and tested for raw spectra as well as the real database. To reduce the probability of false peptide identification, only peptides with significance scores (geq20) at the 99% confidence interval by a Mascot probability analysis greater than “identity” were counted as identified. And each confident protein identification involves at least one unique peptide. For protein quantitation, it was required that a protein contains at least two unique peptides. The quantitative protein ratios were weighted and normalized by the median ratio in Mascot. We only used ratios with *P*-values < 0.05, and only fold changes of >1.2 was considered as significant. Functional annotations of the proteins were conducted using Blast 2 GO program against the non-redundant protein database (NR; NCBI). The Kyoto Encyclopedia of Genes and Genomes (KEGG) database^1^ and the Cluster of Orthologous Groups of proteins (COG) database^2^ were used to classify and group these identified proteins. Gene ontology (GO) is an international standardization of gene function classification system, and it provides a set of dynamic updating controlled vocabulary to describe genes and gene products attributes in the organism. GO has three ontologies, which can describe molecular function, cellular component, biological process, respectively. COG is the database for protein orthologous classification. Proteins in COG are grouped by common ancestry. KEGG pathway is a collection of manually drawn pathway maps representing our knowledge on the molecular interaction and reaction networks. Molecules are represented as nodes, and the biological relationship between two nodes is represented as an edge.

### Expression Analysis by Real-time Polymerase Chain Reaction (PCR)

The expression levels of a gene in multiple organs of both healthy and pathogen-challenged crabs were analyzed by real-time quantitative PCR using SYBR green. The procedure was done according to the published method described previously (17). And the primers were shown in Table S1 in Supplementary Material.

### Prokaryotic Expression and Purification of dsRNA

The primers (shown in Table S1 in Supplementary Material) with specific restriction sites (*Hin*dIII in the forward primer and *Bam*HI in the reverse primer) were designed from the cloned nucleotide sequence. PCR product digested with *Hin*dIII/*Bam*HI was subcloned into LITMUS 38i Vector (New England Biolabs, UK) digested with the same enzymes. The constructed plasmid was verified by restriction enzyme digestion [TLP, anti-lipopolysaccharide factor isoform 4 (ALF iso4), and myosin are *Bam*HI/*Hin*dIII; SCP, β-actin, and anti-lipopolysaccharide factor 4 (ALF 4) are *Eco*RI/*Sal*I]. The recombinant plasmid was transformed into HT115 (DE3) *E. coli* strain. Single colonies of the above the engineering bacteria were separately inoculated to 5 mL of LB medium containing Amp (100 µg/mL), cultured at 37°C with shaking at 200 rpm for 12–16 h, and then inoculated to LB medium containing Amp by a proportion of 1%, cultured at 37°C with shaking at 200 rpm for 2–3 h (OD600 = 0.6), and added with IPTG (with a final concentration of 0.8 mM) to induce the expression for 4 h. Then the dsRNAs were extracted and purified from the bacteria.

### Statistical Analysis

Quantitative data were expressed as mean ± SD. The statistical differences were estimated by one-way analysis of variance followed by least-significant differences and Duncan’s multiple range test. All statistical analyses were carried out using SPSS Statistics version 19. A probability level of 0.01 was used to indicate statistical significance (*P* < 0.01).

## Results

**Figure 1:**
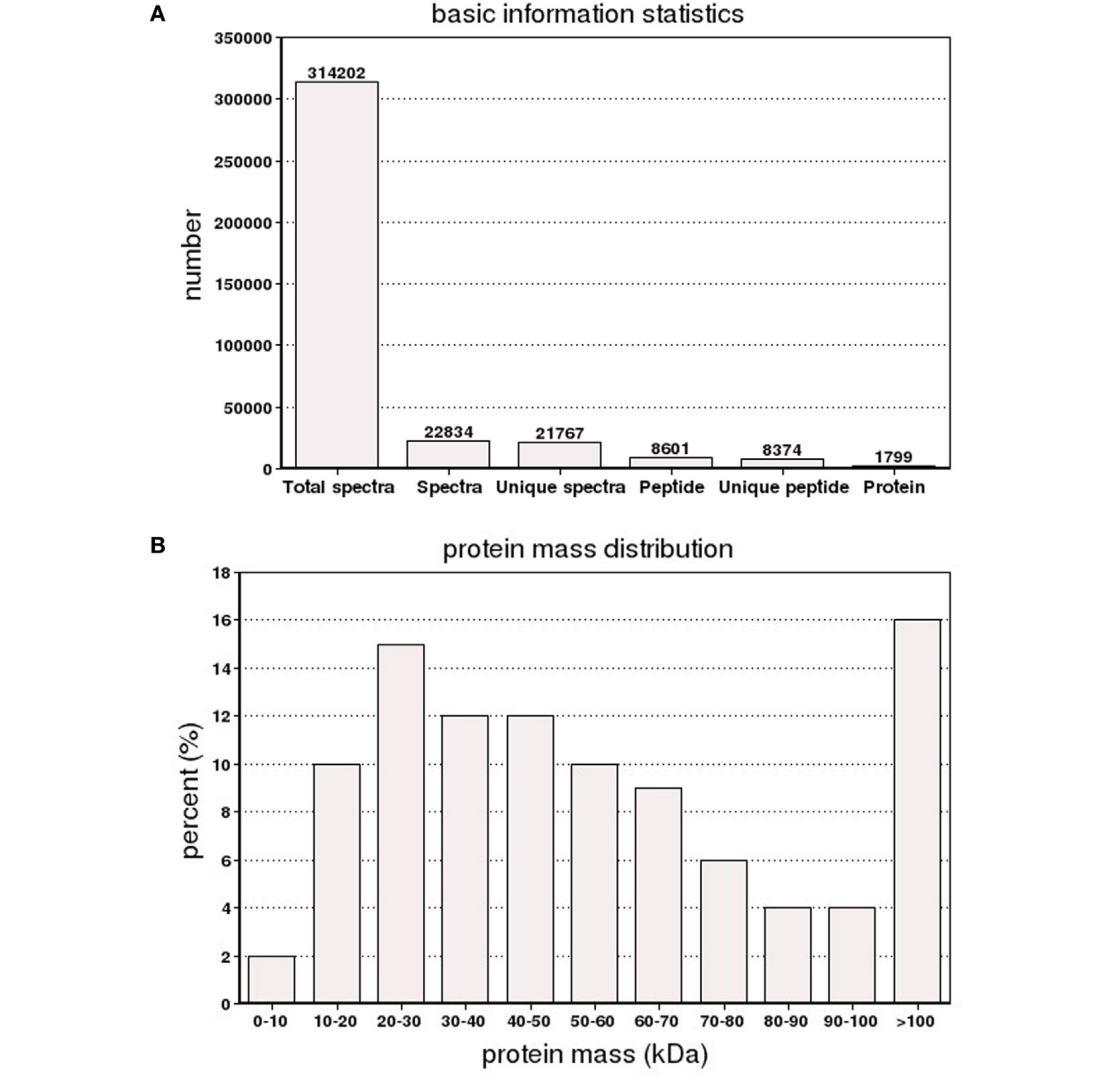

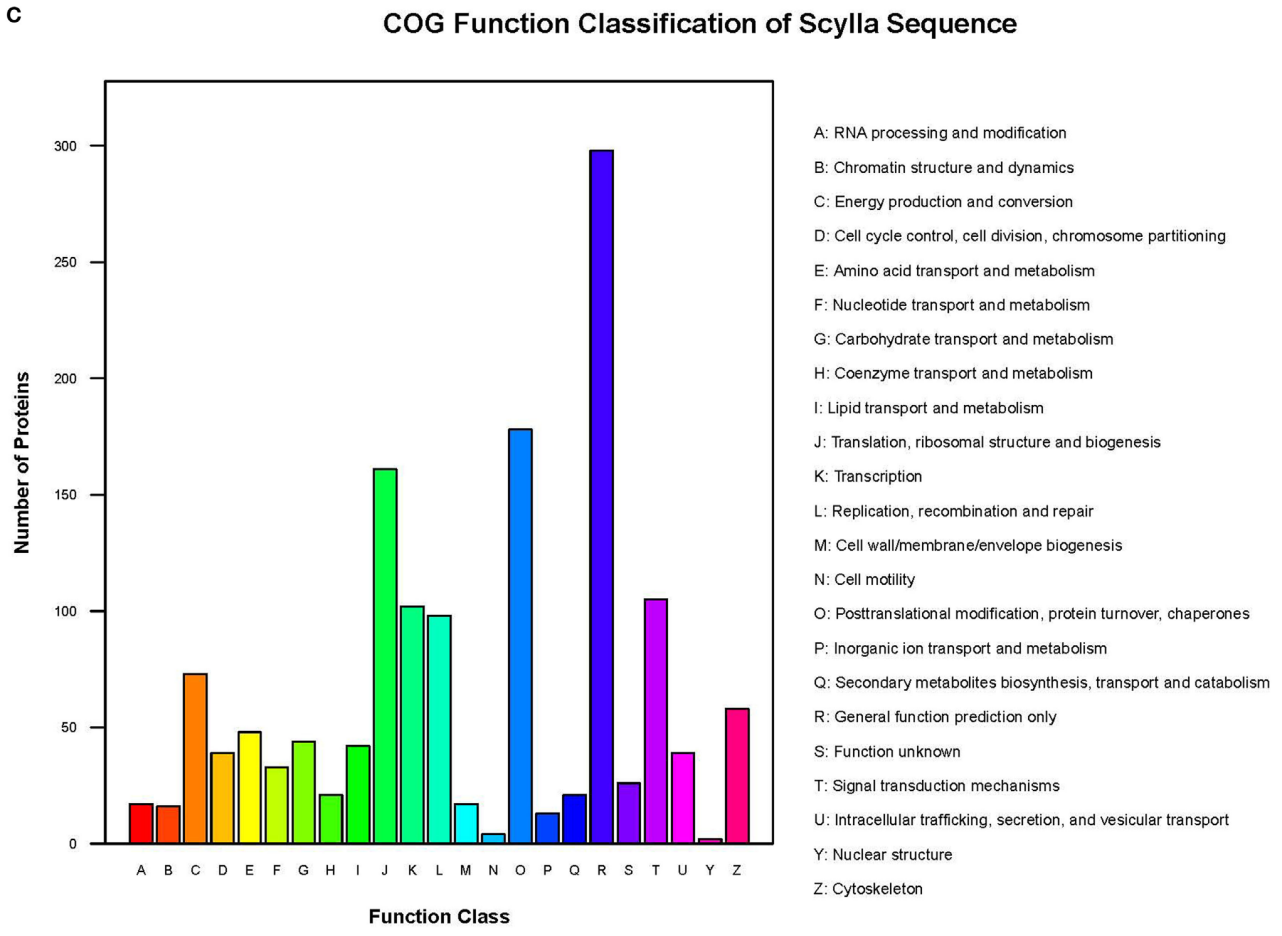
**The basic information and statistics for the isobaric tags for relative and absolute quantitation analysis of *Scylla paramamosain* hemocyte proteome**. **(A)** Coverage of proteins by the identified peptides. **(B)** Distribution of identified proteins among different molecular weights (in kilodaltons). **(C)** Distribution of proteins containing different number of identified peptides.

**Figure 2:**
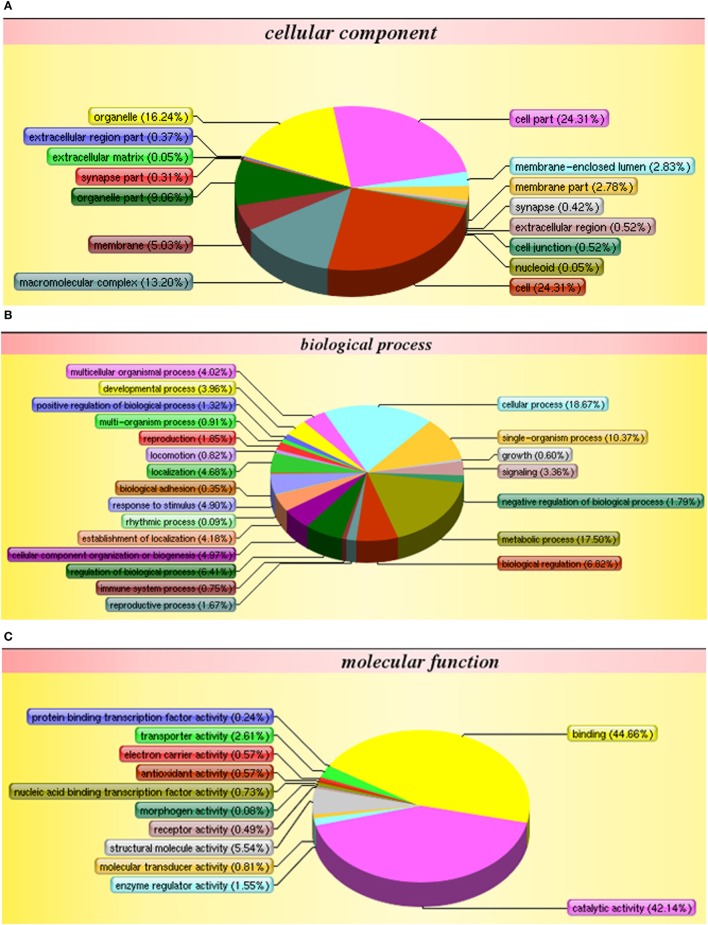

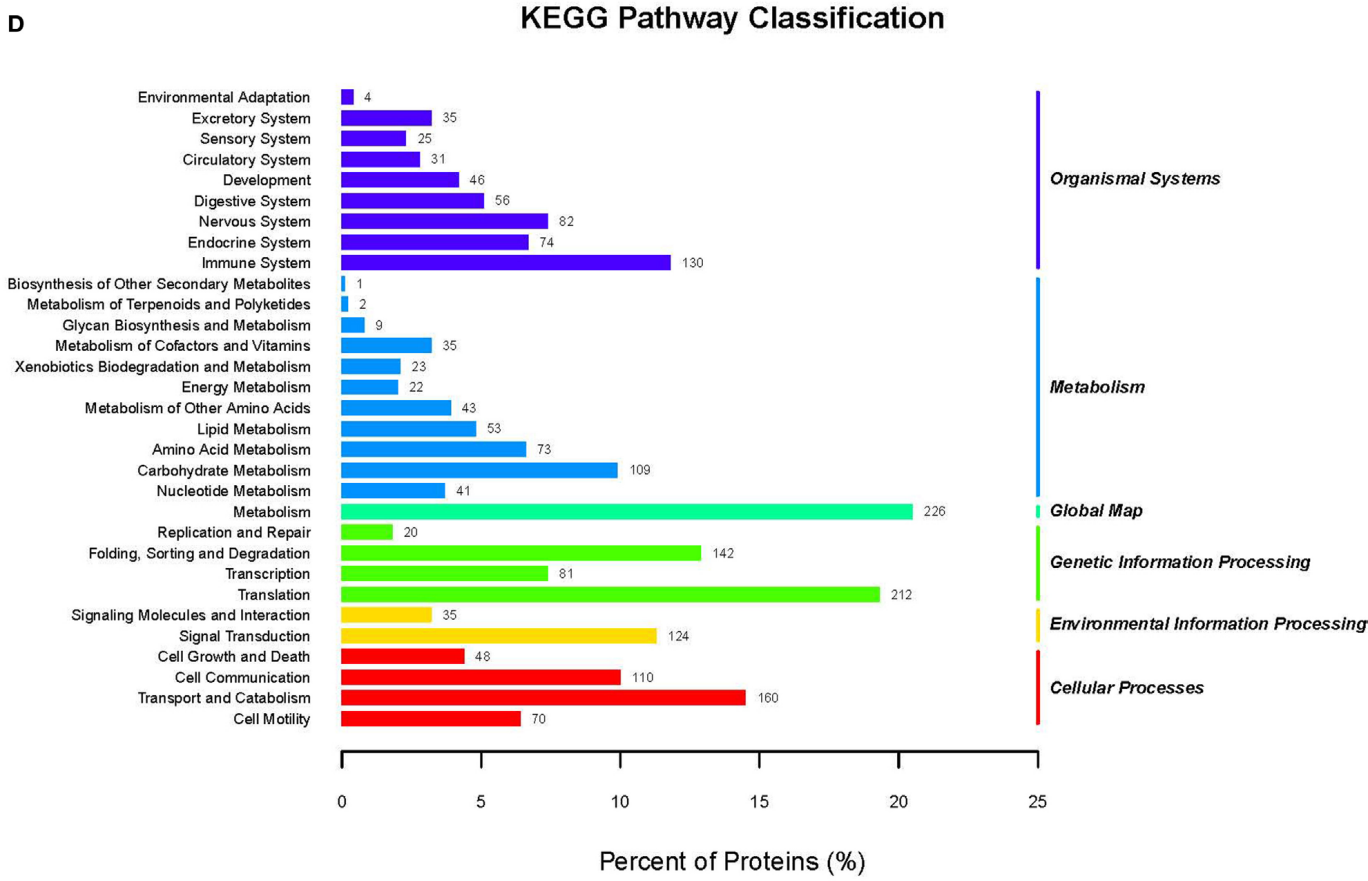
**Gene ontology analysis of proteins in hemocytes of healthy crabs based on cellular component (A), biological process (B), molecular function (C), and Kyoto Encyclopedia of Genes and Genomes (KEGG) pathway classification (D)** of proteins in the hemocytes of healthy crabs.

The iTRAQ analysis of *S. paramamosain* hemocytes proteome showed 22, 834 queries in the database (314,202 sequences) and 1,799 proteins were identified from LC–MS/MS spectra and peptides (Figure 1A). The protein mass distribution showed that protein mass above 100 kDa occupied 16% and protein mass between 10 and 60 kDa occupied 59% (Figure 1B) of the identified proteome. In an effort to analyze the functional distribution of the identified proteins, the known proteins from *S. paramamosain* hemocytes were used as a reference in the COG database (Figure 1C). The functional classification of the *S. paramamosain* sequences showed that the most common function was R: general function prediction only, which indicated that many sequences had an unknown function. The second function was O: posttranslational modification, protein turnover, chaperones, the third function was J: translation, ribosomal structure, and biogenesis. KEGG pathway classification showed that the major organismal system was the metabolism that contained 226 identified proteins, and the major metabolic pathway was carbohydrate metabolism (109 proteins). The second pathway was translation (212 proteins), which represents genetic information processing, and the third pathway was transport and catabolism (160 proteins), which represents cellular processes. The cellular component identified by GO analysis is depicted in Figure 2A. GO analyses showed that the peptides can be categorized into several biological process, i.e., cellular process (18.67%), metabolic process (17.50%), single-organism process (10.37%), and the following two regulatory stages: biological regulation (6.82%) and regulation of biology processes (6.41%) (Figure 2B). The major molecular functions identified by GO analysis of the peptides were binding (44.66%) and catalytic activity (42.14%) (Figure 2C). The KEGG pathway classification of proteins in hemocytes from healthy crabs showed that metabolic pathways and translation are very important (Figure 2D).

**Figure 3:**
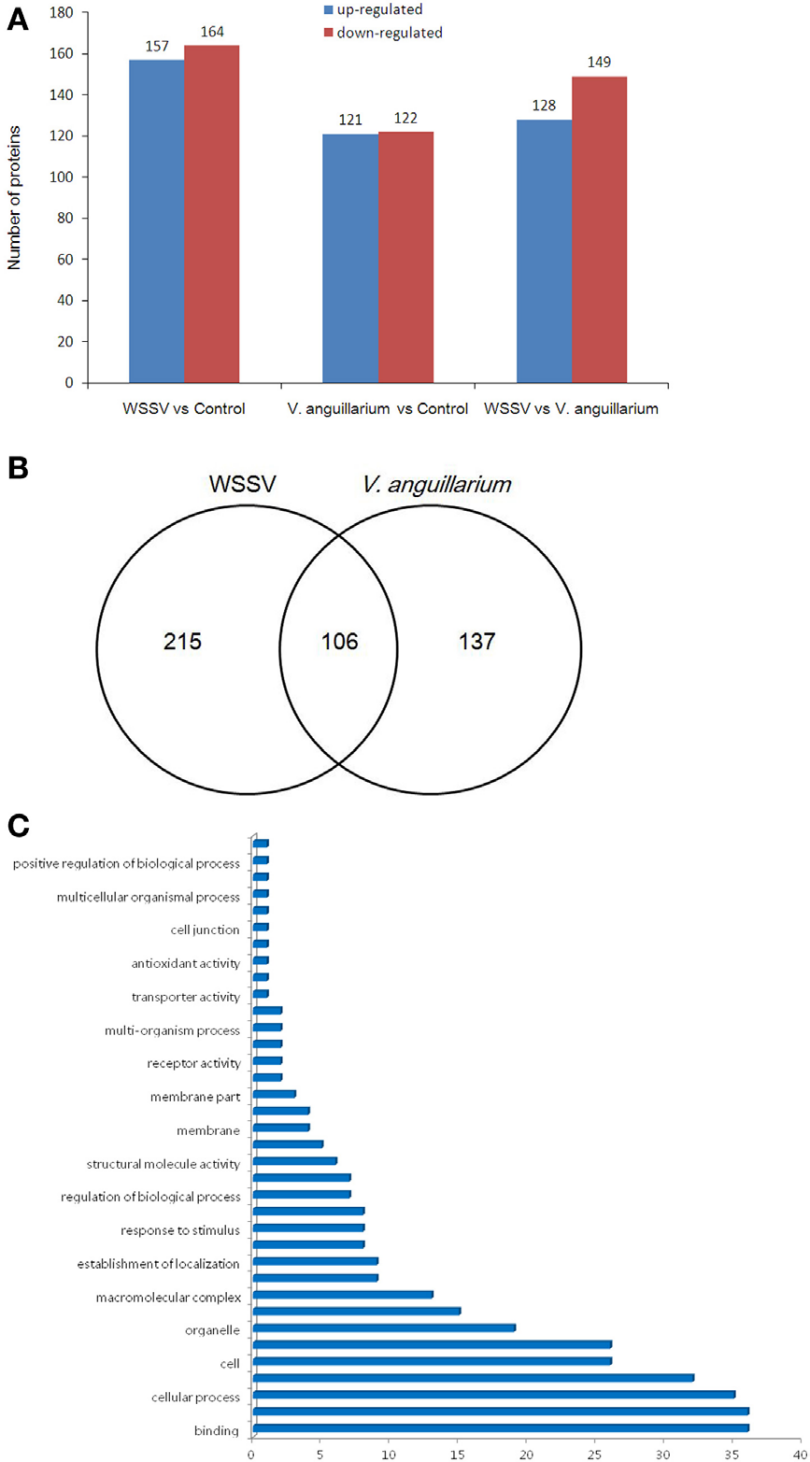
**The differentially expressed proteins were evaluated by isobaric tags for relative and absolute quantitation analysis**. The numbers represent the proteins that were upregulated or downregulated (more than 1.2-fold) compared with the control or *Vibrio alginolyticus* group **(A)**. Venn diagram of differentially expressed genes **(B)**. The numbers represented the proteins upregulated or downregulated (more than 1.2-fold) compared with the control. The statistics of gene ontology enrichment of the 106 proteins up- or downregulated significantly in white spot syndrome virus (WSSV) and *V. alginolyticus* infection **(C)**.

The crabs were challenged with WSSV or *V. alginolyticus*, and then the samples were used for iTRAQ analysis and compared with the control group. We applied a 1.2-fold (*P* < 0.05) increase or decrease as a threshold for significant differences in protein expression to qualify a physiologically important change, 321 proteins were shown to meet this criteria for differential expression, including 157 upregulated proteins and 164 downregulated proteins after WSSV infection (Figures 3A,B). A further 243 proteins showed a 1.2-fold (*P* < 0.05) increase or decrease in protein expression during *V. alginolyticus* infection including, 121 upregulated proteins and 122 downregulated proteins. WSSV infection affected more protein expression in crab hemocytes than *V. alginolyticus* infection. Interestingly, 106 differentially expressed proteins were common to both infections (Figure 3B). Of these, copine-8, troponin C isoform 2b, and sarcoplasmic calcium-binding protein 1 were upregulated significantly, and Sacsin, ALF 4, ALF iso4, and *Pseudomonas aeruginosa* pathogenicity island (PAPI) I were downregulated significantly (Tables 1–3). The data indicate that these proteins may participate in the immune response to WSSV infection and *V. alginolyticus* infection. These 106 proteins can be categorized into some important pathways including carbon metabolism, ribosome, mitogen-activated protein kinase (MAPK) signaling pathway, Ras signaling pathway, phagosome, hippo signaling pathway among others (data not shown). The major molecular functions determined by GO analysis for these proteins were binding, catalytic activity, and organelle components (Figure 3C).

**Table 1:** **The differential expressed proteins in *Scylla paramamosain* hemocytes with above two-fold change post injection with white spot syndrome virus**.

Protein name	Accession	Score	Coverage	Peptide	Fold change
**Upregulated proteins**
Copine-8	comp20718_c0_seq2_No.1	841	30.9	14	6.29
Troponin C isoform 2b	comp22592_c0_seq1_No.1	69	26.3	3	5.35
Sarcoplasmic calcium-binding protein 1	comp40442_c0_seq1_No.1	83	16.1	3	4.78
β-Actin	comp12583_c0_seq1_No.1	1,377	67.2	4	4.02
Myosin light chain 2	comp21132_c0_seq1_No.1	765	34.1	9	3.58
Slow muscle myosin S1 heavy chain	comp20546_c0_seq16_No.1	2,058	43.7	26	2.69
clathrin light chain	comp17823_c1_seq1_No.1	147	22.5	4	2.67
Ubiquitin carboxyl-terminal esterase L3	comp16837_c1_seq1_No.1	396	31.6	6	2.65
2-Phospho-d-glycerate hydrolase	comp20783_c0_seq1_No.1	1,685	50.7	18	2.65
Membrane-associated protein	comp51893_c0_seq1_No.1	281	15.4	2	2.64
Alpha actin	comp12775_c1_seq1_No.1	1,274	57	5	2.56
Spermatogonial stem-cell renewal factor	comp72221_c0_seq1_No.1	240	41.9	9	2.48
Inorganic pyrophosphatase-like protein	comp12817_c0_seq1_No.1	216	25.3	5	2.44
Putative phosphoglycerate kinase	comp12041_c0_seq1_No.1	897	33.5	13	2.4
Protein kinase c	comp18689_c0_seq1_No.1	185	13.4	8	2.36
Glutathione *S*-transferase	comp15053_c0_seq1_No.1	1,634	50.5	10	2.19
Myosin-9	comp12936_c0_seq1_No.1	382	18.3	13	2.1
Myosin heavy chain type 1	comp20546_c0_seq7_No.1	186	38.8	2	2.04
**Downregulated proteins**
Sacsin	comp19443_c0_seq1_No.1	42	0.2	1	8.76
Pacifastin-like serine protease inhibitor	comp3991_c0_seq1_No.1	82	15.1	2	5.45
Male reproductive tract-specific Kazal-type proteinase inhibitor	comp19421_c0_seq1_No.1	164	11.7	1	3.77
*Pseudomonas aeruginosa* pathogenicity island I	comp20408_c0_seq1_No.1	54	7.6	1	3.1
Anti-lipopolysaccharide factor isoform 5	comp12264_c0_seq1_No.1	179	27.6	2	2.59
Putative protein phosphatase	comp18826_c0_seq1_No.1	117	16.4	2	2.52
Crustin antimicrobial peptide	comp12292_c0_seq1_No.1	462	35	2	2.47
Glutathione-dependent prostaglandin D synthase	comp16103_c0_seq1_No.1	133	25.1	5	2.42
Cuticular protein analogous to peritrophins 3	comp12594_c0_seq1_No.1	147	9.3	2	2.4
60S acidic ribosomal protein P2-like protein	comp21230_c0_seq1_No.1	292	66.7	5	2.2
Glutathione peroxidase 6 precursor	comp10211_c0_seq1_No.1	157	17.7	4	2.18
Chain A, crystal structure of Chmp4b hairpin	comp21559_c0_seq1_No.1	356	35.3	5	2.15
Calreticulin precursor	comp14413_c0_seq1_No.1	194	15.8	6	2.12
RIKEN	comp2503_c0_seq1_No.1	47	11.3	2	2.08
Lactate dehydrogenase	comp19912_c0_seq3_No.1	87	16.9	5	2.03
Heat shock protein 90	comp10311_c0_seq1_No.1	332	18.5	12	2

**Table 2:** **The differential expressed proteins in *Scylla paramamosain* hemocytes with above two-fold change post injection with *V. anguillarum***.

Protein name	Accession	Score	Coverage	Peptide	Fold change
**Upregulated proteins**
Sarcoplasmic calcium-binding protein 1	comp40442_c0_seq1_No.1	83	16.1	3	8.40
Troponin C isoform 2b	comp22592_c0_seq1_No.1	69	26.3	3	2.78
Clip domain serine proteinase 3	comp19522_c0_seq1_No.1	520	33.8	9	2.40
Copine-8	comp20718_c0_seq2_No.1	841	30.9	14	2.19
Slow muscle myosin S1 heavy chain	comp20546_c0_seq16_No.1	2,058	43.7	26	2.13
Putative secreted salivary gland peptide	comp12272_c1_seq1_No.1	83	22	3	2.23
Chloride intracellular Channel isoform 1	comp20820_c0_seq1_No.1	100	25.7	5	2.11
Myophilin	comp18304_c1_seq2_No.1	1,662	62.4	8	2.03
Spermine synthase	comp18763_c0_seq1_No.1	200	16.1	4	2.02
Putative phosphoglycerate kinase	comp12041_c0_seq1_No.1	897	33.5	13	2.02
**Downregulated proteins**
Sacsin	comp19443_c0_seq1_No.1	42	0.2	1	31.39
Phospholipase D1	comp1157_c0_seq1_No.1	211	17.4	5	6.97
Low-density lipoprotein receptor	comp20609_c0_seq1_No.1	283	10.2	6	5.77
*Pseudomonas aeruginosa* pathogenicity island I	comp20408_c0_seq1_No.1	54	7.6	1	3.02
pacifastin-like serine protease inhibitor	comp3991_c0_seq1_No.1	82	15.1	2	2.99
Anti-lipopolysaccharide factor 4	comp12264_c0_seq1_No.1	179	27.6	2	2.82
Anti-lipopolysaccharide factor isoform 4	comp20686_c1_seq1_No.1	126	47.7	4	2.12

**Table 3:** **The selected proteins in *Scylla paramamosain* hemocytes with over two-fold change post infection**.

Protein name	Accession	Score	Coverage	Peptide	Fold change	
					White spot syndrome virus	VA
**Cytoskeleton/extracellular proteins**
β-Actin	comp12583_c0_seq1_No.1	1,377	67.2	4	+4.02	–1.23
Myosin-9	comp12936_c0_seq1_No.1	382	18.3	13	+2.11	+1.96
**Immunologic proteins**
Anti-lipopolysaccharide factor 4	comp12264_c0_seq1_No.1	179	27.6	2	–2.59	–2.82
Anti-lipopolysaccharide factor isoform 4	comp20686_c1_seq1_No.1	126	47.7	4	–1.86	–2.12
**Physiologic proteins**
Transketolase-like protein 2-like isoform 1	comp18784_c0_seq2_No.1	3,418	44.3	21	–2.19	–2.2
Sarcoplasmic calcium-binding protein 1	comp40442_c0_seq1_No.1	83	16.1	3	+4.78	+8.4

**Figure 4:**
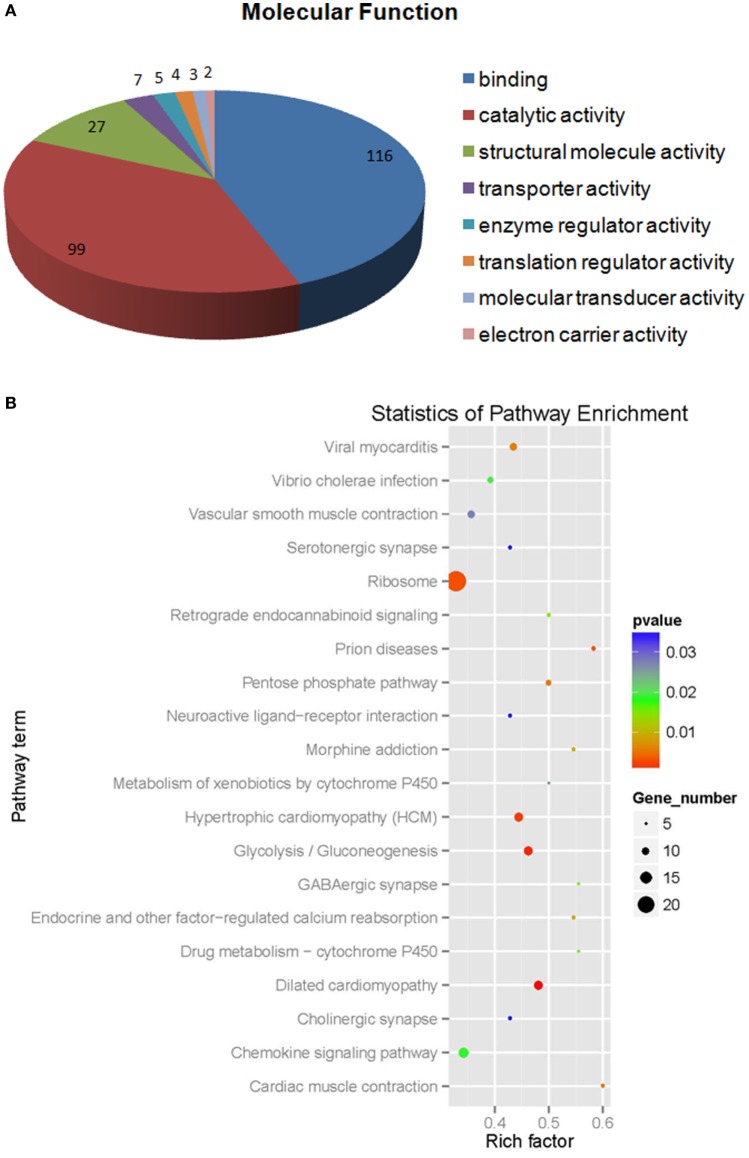
**The molecular function (A) statistics of pathway enrichment (B) of differential expressed proteins (more than 1.2-fold) from white spot syndrome virus group vs control group**.

Of the differentially expressed proteins in WSSV infection, the GO enrichment scatterplot showed that the organelle and intracellular organelle proteins were the most important, and a KEGG enrichment scatterplot indicated that metabolic pathways were the most important (Figure 4; Figure S1A in Supplementary Material). The differentially expressed proteins (over twofold) and the related iTRAQ data are presented in Table 1. The upregulated proteins included copine-8 with 6.29-fold change, troponin C isoform 2b with 5.35-fold change, sarcoplasmic calcium-binding protein 1 with 4.78-fold change, beta-actin with 4.02-fold change, myosin light chain 2 with 3.58-fold change, slow muscle myosin S1 heavy chain with 2.69-fold change, clathrin light chain with 2.67-fold change, ubiquitin carboxyl-terminal esterase L3 with 2.65-fold change, 2-phospho-d-glycerate hydrolase with 2.65-fold change, membrane-associated protein with 2.64-fold change, alpha actin with 2.56-fold change, myosin-9 with 2.1-fold change, myosin heavy chain type 1with 2.04-fold change and some enzymes with above a twofold change (Table 1). The data indicate that many endocytosis-related cytoskeleton proteins of host hemocytes contribute to WSSV infection. The upregulated proteins and the related iTRAQ data are presented in Supplementary Material Table S1. The downregulated proteins included Sacsin with 8.76-fold change, pacifastin-like serine protease inhibitor with 5.45-fold change, male reproductive tract-specific Kazal-type proteinase inhibitor with 3.77-fold change, PAPI-1 with 3.1-fold change, ALF 4 with 2.59-fold change, crustin AMP with 2.47-fold change, heat shock protein 90 with twofold change and some enzymes with above a twofold change.

**Figure 5:**
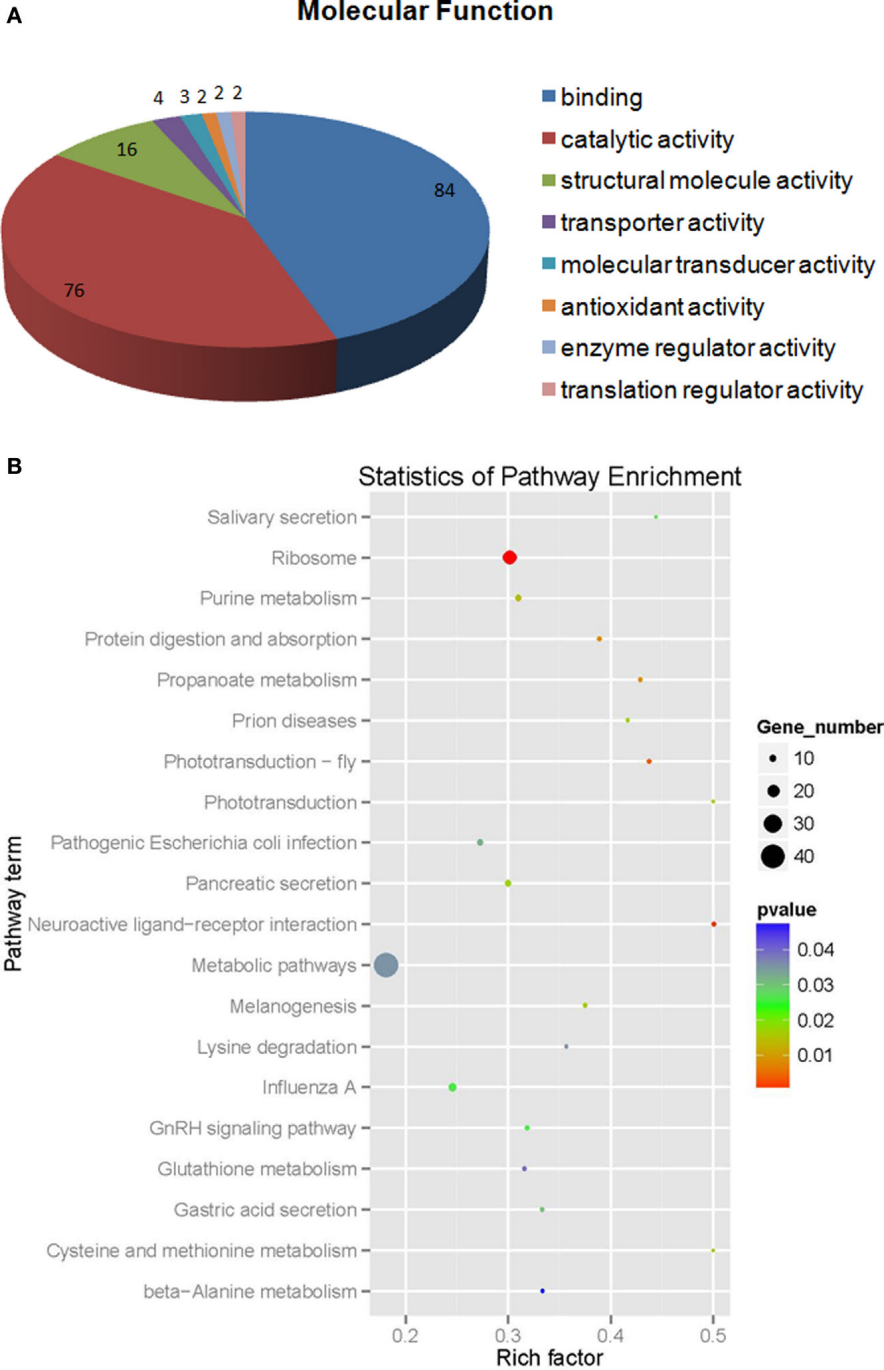
**The molecular function (A) statistics for pathway enrichment (B) of differentially expressed proteins (more than 1.2-fold) from *Vibrio alginolyticus* vs control**.

Of the differentially expressed proteins in *V. alginolyticus* infection, the GO enrichment scatterplot showed that the organelle and intracellular organelle are the most important, and a KEGG enrichment scatterplot indicated that metabolic pathways were the most important (Figure 5; Figure S1B in Supplementary Material). The differentially expressed proteins (over twofold) and the related iTRAQ data are presented in Table 2. The upregulated proteins included sarcoplasmic calcium-binding protein 1 with 8.4-fold change, troponin C isoform 2b with 2.78-fold change, clip domain serine proteinase 3 with 2.4-fold change, putative secreted salivary gland peptide with 2.23-fold change, copine-8 with 2.17-fold change, slow muscle myosin S1 heavy chain with 2.13-fold change, chloride intracellular channel isoform 1 with 2.11-fold change, and myophilin with 2.03-fold change (Table 2). The downregulated proteins included Sacsin with 31.39-fold change, phospholipase D1 with 6.97-fold change, low-density lipoprotein receptor with 5.77-fold change, PAPI I with 3.02-fold change, pacifastin-like serine protease inhibitor with 2.99-fold change, ALF 4 with 2.82-fold change and ALF iso4 with 2.12-fold change. It was showed that intracellular non-membrane-bounded organelle is the most important in statistics of GO enrichment of differentially expressed proteins (more than 1.2-fold) from WSSV vs *V. alginolyticus*, and phagosome is the most important in statistics of GO enrichment (Figure S2 in Supplementary Material).

**Figure 6:**
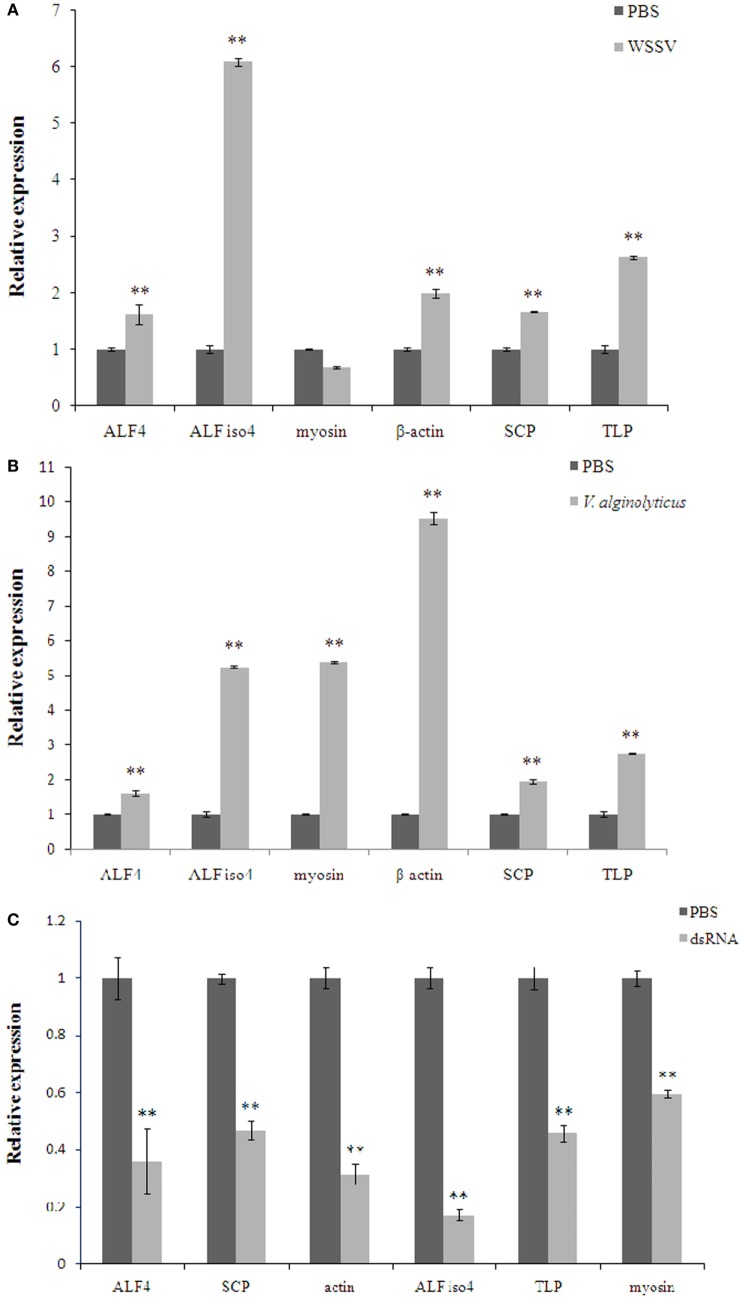
**The expression of the selected genes in *Scylla paramamosain* hemocytes in response to white spot syndrome virus (WSSV) (A), *Vibrio alginolyticus* (B), and specific dsRNA (C)**. Data are shown as means ± SD of three separate individuals in the tissues. Capital letters indicate expression of crustin-like different adult tissues. Double asterisks indicate a significant difference (*P* < 0.01) between two samples. ALF 4, anti-lipopolysaccharide factor 4; ALF iso4, anti-lipopolysaccharide factor isoform 4; myosin-9, myosin; TLP, transketolase-like protein 2-like isoform 1; SCP, sarcoplasmic calcium-binding protein 1.

Interestingly, some proteins were changed significantly (*P* < 0.01) in WSSV and *V. alginolyticus* infection (Table S2 in Supplementary Material; Table 3). Six differentially expressed genes, β-actin, myosin-9, ALF iso4, ALF 4, transketolase-like protein 2-like isoform 1, and sarcoplasmic calcium-binding protein 1 were characterized by real-time PCR assay (Figures 6A,B). The expression of these six genes showed a trend of upregulation at 24 h post WSSV or *V. alginolyticus* challenge except for that of myosin-9 in response to WSSV. Only β-actin and sarcoplasmic calcium-binding protein 1 showed the same trend in the proteomic results. dsRNA silencing results showed that when the dsRNA was present expression of these six genes was significantly decreased (*P* < 0.01) (Figure 6C).

**Figure 7:**
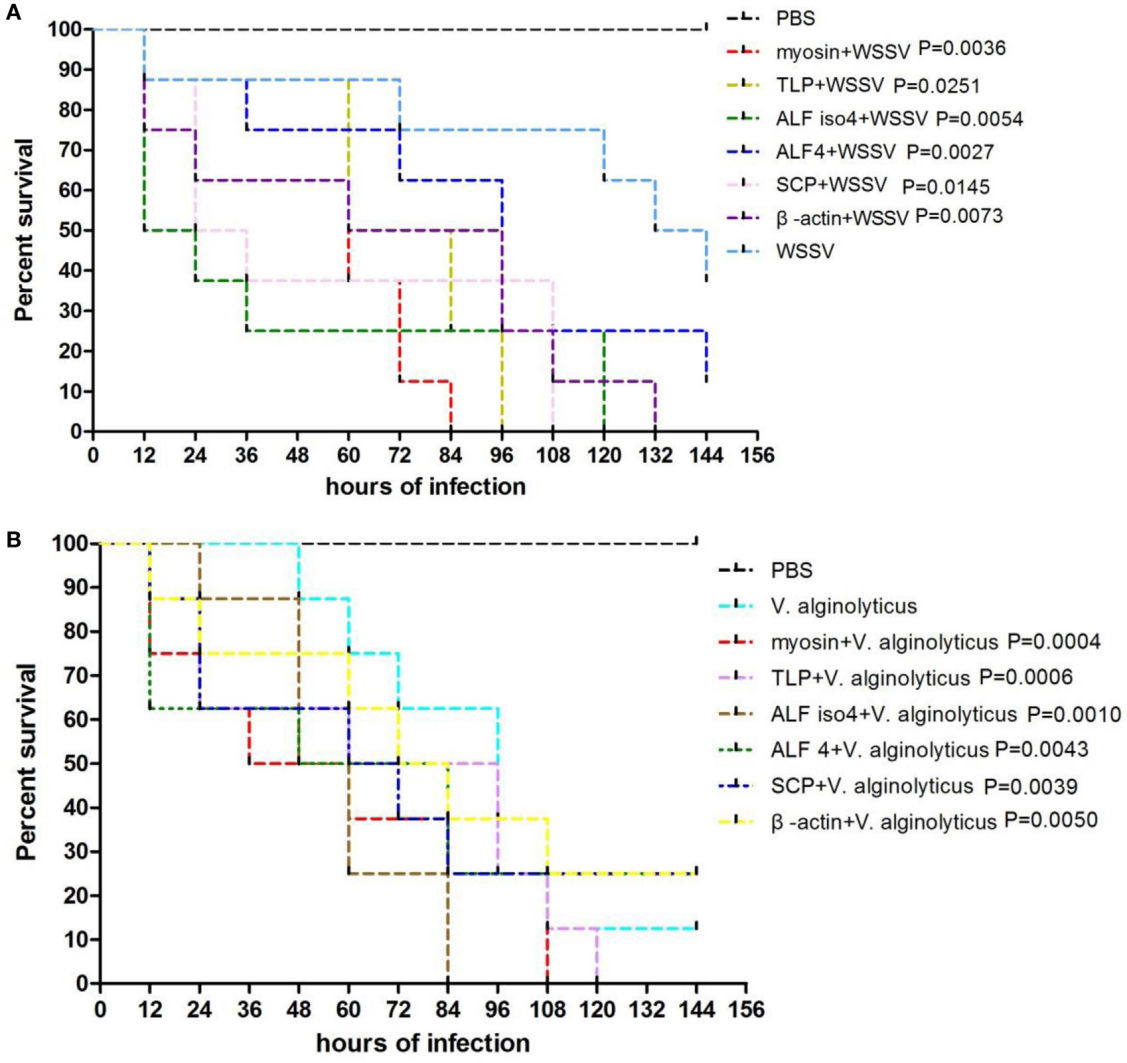
**Comparison of protective effects between six selected genes**. The percentage survival of challenged crabs treated with dsRNA. Juvenile *Scylla paramamosain* were injected intramuscularly with dsRNAs 24 h before white spot syndrome virus (WSSV) challenge **(A)** or *Vibrio alginolyticus* challenge **(B)**. Control group was injected with phosphate-buffered saline (PBS) solution, and WSSV or *V. alginolyticus* group was challenged by WSSV or *V. alginolyticus*. The treatment is shown on the right. The *P*-value is the data of experimental groups compared to that of WSSV or *V. alginolyticus* group.

To confirm the protective effects of these six genes, *S. paramamosain* were injected intramuscularly with specific dsRNAs before WSSV or *V. alginolyticus* challenge. There were 16 crabs in each group and experiments were conducted for 144 hours. The control group was injected with PBS solution followed by WSSV challenge. Each point represents the means of a triplicate assays within the SD. Then we tested the mortality rates of crabs when they were infected by WSSV or *V. alginolyticus*. The data showed that the knockdown of these six genes led to a faster morbidity rate in the WSSV-infected crabs and significantly (*P* < 0.01) increased the mortality of crabs compared with that of the control WSSV group (Figure 7A). In the first 84 h, knockdown of these six genes also led to a faster morbidity rate in the *V. alginolyticus*-infected crabs but did not change the mortality rate significantly when compared with that of the *V. alginolyticus* control group (Figure 7B). However, four genes did show a higher mortality rate than that of the control after they were knocked down.

## Discussion

In this study, we identified 1,799 proteins through the iTRAQ analysis of the *S. paramamosain* hemocytes proteome and showed that proteins with a mass between 10–60 kDa occupied 59% of all proteins in these animals. The major pathways included metabolism, 226 proteins, translation, 212 proteins, and transport and catabolism, 160 proteins. The important biological processes included cellular and metabolic processes, and the major molecular functions were binding and catalytic activity. No matter for WSSV or *V. alginolyticus* infection, the organelle and intracellular organelle are the most important, and metabolic pathways were the key pathways. WSSV was found to induce metabolic changes (17), and endocytosis is vital for its replication in crustacean (18). *V. alginolyticus* infection would damage the mitochondria of hemocytes and then cause hemocyte death in crabs (19). Interestingly, we found that WSSV infection significantly affected more proteins’ expression in host hemocytes than *V. alginolyticus* infection. Many cytoskeleton proteins contribute to WSSV infection indicating that phagocytosis is the most important cellular process during infection. This finding is accordance with previous reports (11, 20). Interestingly, we found that 106 proteins were differentially expressed simultaneously in WSSV infection and *V. alginolyticus* infection. These proteins can be categorized into some important pathways including carbon metabolism, ribosome, MAPK signaling pathway, Ras signaling pathway, phagosome, and Hippo signaling pathway. The major molecular functions of these proteins were binding, catalytic activity, and organelle composition.

Then, we chose six differentially expressed genes to investigate their expression and the protective effects against WSSV infection or *V. alginolyticus* infection. We found that these six genes showed a trend of upregulation at 24 h post-WSSV or *V. alginolyticus* challenge except for that of myosin-9 in response to WSSV, and only β-actin and sarcoplasmic calcium-binding protein 1 showed the same trend in the proteomic results. We found that the knockdown of these six genes led to an increase in morbidity and mortality in the WSSV-infected crabs when compared with that of WSSV control group. Similarly, knockdown of these six genes also led to an increased morbidity rate in the *V. alginolyticus*-infected crabs in the first 84 h and four genes showed an increase in the mortality rate when compared to the *V. alginolyticus* control group. β-Actin and myosin are known to regulate phagocytosis in shrimp and participate in the response to WSSV (11, 20, 21). Phagocytosis is also the key immune response to bacterial challenge in invertebrates (22). In this study, β-actin and myosin-9 were upregulated significantly (*P* < 0.01) in WSSV infection, but β-actin was downregulated and myosin-9 upregulated in *V. alginolyticus* infection. When β-actin or myosin-9 was knocked down during WSSV or *V. alginolyticus* infection, there was an increase in the morbidity and mortality rates. The data indicated that β-actin and myosin-9 play an important role in the immune response to WSSV or *V. alginolyticus* infection.

Sarcoplasmic calcium-binding protein 1 is believed to function as the invertebrate equivalent of vertebrate parvalbumin, namely, to “buffer” cytosolic Ca^2+^, and its expression is downregulated in pre- and postmolt stages compared with intermolt in crayfish (23). Sarcoplasmic calcium-binding protein is also an important allergen in the muscle and shell of *Fenneropenaeus merguiensis* (24). WSSV infection in *Penaeus monodon* is facilitated by housekeeping molecules like sarcoplasmic calcium-binding protein 1 (25). When sarcoplasmic calcium-binding protein 1 was knocked down, WSSV infection would cause the animals to die faster and more often, but in *V. alginolyticus* infection this would only reduce the lifespan of infected crabs but decreased the overall mortality rates. These results revealed that sarcoplasmic calcium-binding protein 1 plays an important role in the immune response to WSSV and plays a different role in the immune response to *V. alginolyticus* infection. The anti-lipopolysaccharide factors (ALFs) are well known for being the most common effectors affecting the invading microorganisms in crustaceans and its antimicrobial activity in killing Gram-positive and -negative bacteria and viruses (26–31). In this study, ALF 4 and ALF iso4 were upregulated significantly in WSSV and *V. alginolyticus* infection. When ALF iso4 was knocked down, in either WSSV or *V. alginolyticus* infection it resulted in a reduced lifespan an increase in the mortality rate. However, the knockdown of ALF 4 leads to increased morbidity and mortality in WSSV-infected crabs and increased morbidity but decreased mortality in *V. alginolyticus*-infected crabs. The transketolase is required for cancer growth because of its ability to affect the production of NAPDH to counteract oxidative stress (32). The transketolase-like protein 2-like isoform 1 was downregulated significantly in WSSV infection and *V. alginolyticus* infection. The knockdown of transketolase-like protein 2-like isoform 1 caused an increase in the morbidity and mortality rates following infection with either pathogen.

In this investigation, we reported on the proteome of the mud crab *S. paramamosain* in response to WSSV or *V. alginolyticus* infection. We investigated the molecular mechanisms underlying the immune response to WSSV infection in crustaceans. These findings will contribute to our understanding of the molecular mechanisms of immune response to WSSV or *V. alginolyticus* infection in crab hemocytes.

## Ethics Statement

The animal subjects used in the present study are crab, which are invertebrates and are exempt from this requirement.

## Author Contributions

FZ and BS designed experiments, analyzed experimental results, and wrote the manuscript; BS, ZhW, ZiW, and XM carried out experiments.

## Conflict of Interest Statement

There are no patents, products in development, or marketed products to declare. This does not alter our adherence to all the gene policies on sharing data and materials.

## Footnotes

^1^http://www.genome.jp/kegg/.

^2^http://www.ncbi.nlm.nih.gov/COG/.

## Supplementary Material

Figure S1 **(A)** Kyoto Encyclopedia of Genes and Genomes (KEGG) pathway classification of differential expressed proteins (more than 1.2-fold) from white spot syndrome virus group vs control group. **(B)** KEGG pathway classification of differentially expressed proteins (more than 1.2-fold) from *Vibrio alginolyticus* vs control.

Figure S2 The statistics of gene ontology (GO) enrichment **(A)** and statistics of pathway enrichment **(B)** of differentially expressed proteins (more than 1.2-fold) from white spot syndrome virus vs *Vibrio alginolyticus*.

## Abbreviations

iTRAQ, isobaric tags for relative and absolute quantitation; WSSV, white spot syndrome virus; KEGG, Kyoto Encyclopedia of Genes and Genomes; COG, Cluster of Orthologous Groups of proteins; PCR, polymerase chain reaction; GO, gene ontology; MAPK, mitogen-activated protein kinase; PAPI, *Pseudomonas aeruginosa* pathogenicity island; TEAB, tetraethyl ammonium bromide; ACN, acetonitrile; FA, formaldehyde.
