# Supplementary figures and images for: A Proteomic Study of Hemocyte Proteins from Mud Crab (Scylla paramamosain) Infected with White Spot Syndrome Virus or Vibrio alginolyticus
Source: Front Immunol. 2017 Apr 27;8:468. doi: 10.3389/fimmu.2017.00468 (PMC5406513; doi:10.3389/fimmu.2017.00468)

A

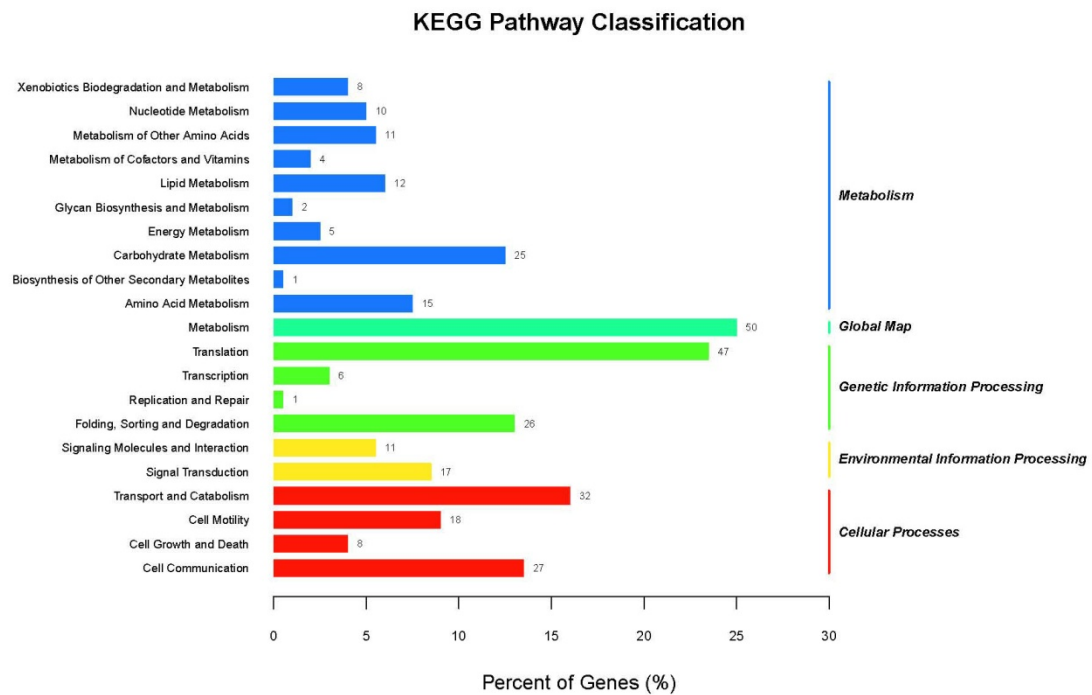

B

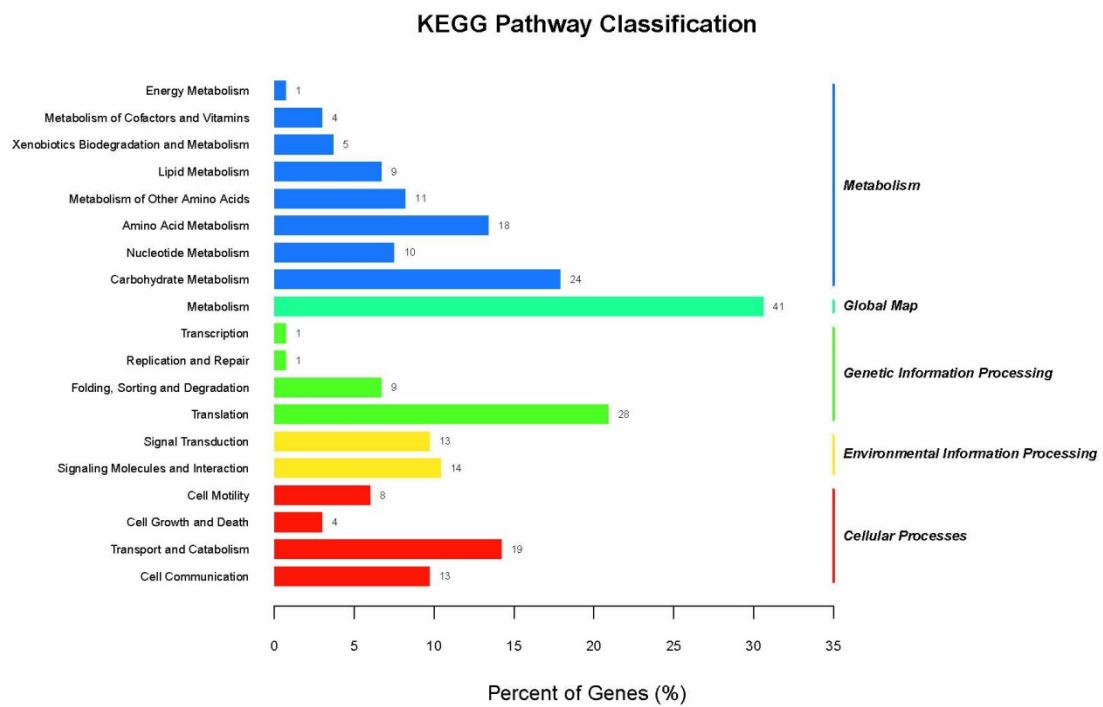

Supplement: Figure S1 — (A) Kyoto Encyclopedia of Genes and Genomes (KEGG) pathway classification of differential expressed proteins (more than 1.2-fold) from white spot syndrome virus group vs control group. (B) KEGG pathway classification of differentially expressed proteins (more than 1.2-fold) from Vibrio alginolyticus vs control. [file image_1.pdf]

A

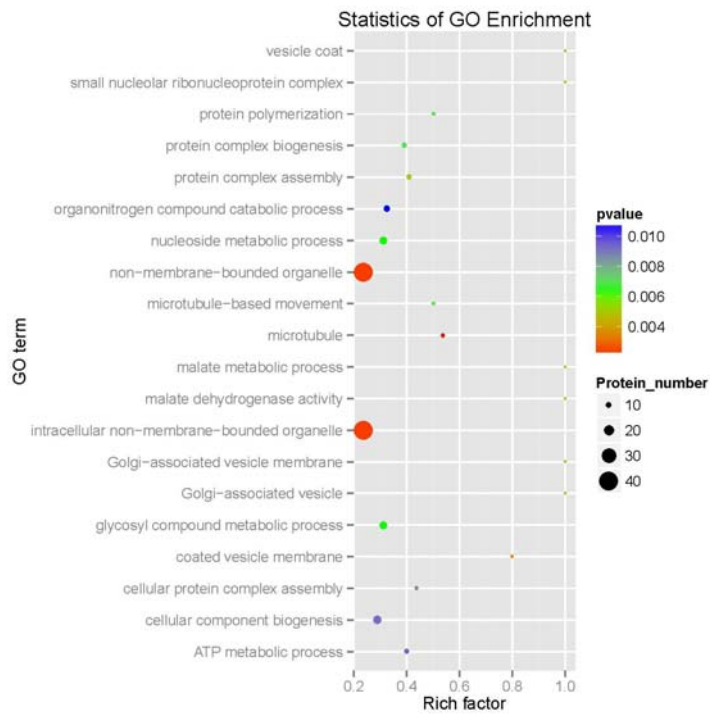

B

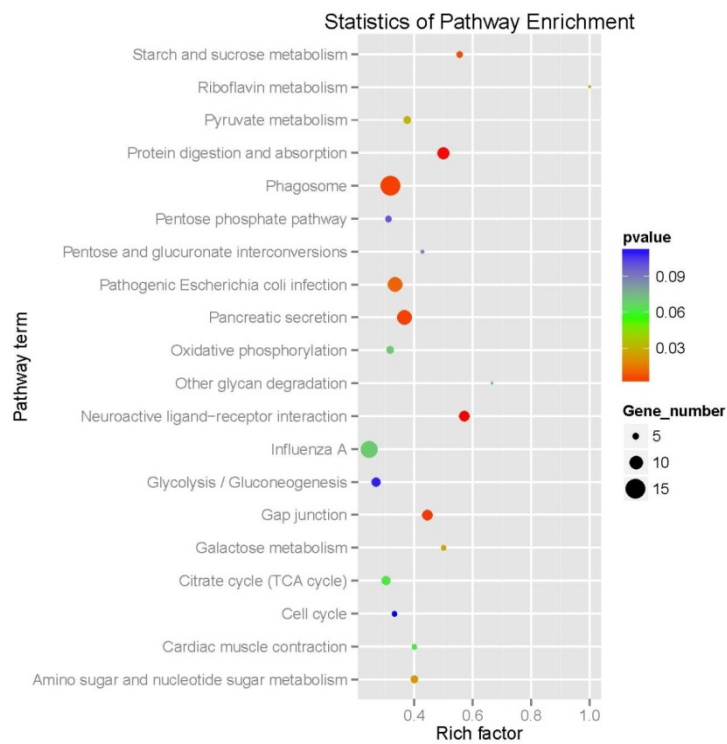

Supplement: Figure S2 — The statistics of gene ontology (GO) enrichment (A) and statistics of pathway enrichment (B) of differentially expressed proteins (more than 1.2-fold) from white spot syndrome virus vs Vibrio alginolyticus. [file image_2.pdf]
