# Supplementary material for: A Proteomic Study of Hemocyte Proteins from Mud Crab (Scylla paramamosain) Infected with White Spot Syndrome Virus or Vibrio alginolyticus
Source: Front Immunol. 2017 Apr 27;8:468. doi: 10.3389/fimmu.2017.00468 (PMC5406513; doi:10.3389/fimmu.2017.00468)
Supplement: Supplementary file 3 [file table_1.docx]

**Table S1. The selected proteins in *Scylla paramamosain* hemocytes with over 2-fold change post infection.**

| Protein name | Accession | Score | Coverage | Peptide | Fold change | |
| --- | --- | --- | --- | --- | --- | --- |
|  |  |  |  |  | WSSV | VA |
| **Cytoskeleton/extracellular proteins** |  |  |  |  |  |  |
| β-actin | comp12583_c0_seq1_No.1 | 1377 | 67.2 | 4 | +4.02 | -1.23 |
| myosin-9 | comp12936_c0_seq1_No.1 | 382 | 18.3 | 13 | +2.11 | +1.96 |
| myosin light chain 2 | comp21132_c0_seq1_No.1 | 765 | 34.1 | 9 | +3.58 | +1.5 |
| low-density lipoprotein receptor | comp20609_c0_seq1_No.1 | 283 | 10.2 | 6 | -1.65 | -5.78 |
| **Immunologic proteins** |  |  |  |  |  |  |
| anti-lipopolysaccharide factor isoform 4 | comp20686_c1_seq1_No.1 | 126 | 47.7 | 4 | -1.86 | -2.12 |
| anti-lipopolysaccharide factor isoform 5 | comp12264_c0_seq1_No.1 | 179 | 27.6 | 2 | -2.59 | -2.82 |
| PAPI I | comp20408_c0_seq1_No.1 | 54 | 7.6 | 1 | -3.10 | -3.02 |
| pacifastin-like serine protease inhibitor | comp3991_c0_seq1_No.1 | 82 | 15.1 | 2 | -5.45 | -2.99 |
| **Physiologic proteins** |  |  |  |  |  |  |
| Sacsin | comp19443_c0_seq1_No.1 | 42 | 0.2 | 1 | -8.76 | -31.39 |
| copine-8 | comp20718_c0_seq2_No.1 | 841 | 30.9 | 14 | +6.29 | +2.17 |
| transketolase-like protein 2-like isoform 1 | comp18784_c0_seq2_No.1 | 3418 | 44.3 | 21 | -2.19 | -2.2 |
| laccase | comp20661_c0_seq1_No.1 | 889 | 26.8 | 17 | -3.29 | -2.32 |
| glutathione S-transferase | comp15053_c0_seq1_No.1 | 1634 | 50.5 | 10 | +2.19 | +1.79 |
| sarcoplasmic calcium-binding protein 1 | comp40442_c0_seq1_No.1 | 83 | 16.1 | 3 | +4.78 | +8.4 |
| troponin C isoform 2b | comp22592_c0_seq1_No.1 | 69 | 26.3 | 3 | +5.35 | +2.78 |
| **Intracellular proteins** |  |  |  |  |  |  |
| 7,8-dihydro-8-oxoguanine-  triphosphatase | comp179853_c0_seq1_No.1 | 53 | 4.1 | 1 | -16.03 | -31.92 |
| mitochondrial glutamate carrier protein | comp11638_c1_seq1_No.1 | 110 | 15.5 | 2 | -5.19 | -13.97 |
| **Unknown/hypothetical proteins** |  |  |  |  |  |  |
| hypothetical proteins | comp9758_c0_seq1_No.1 | 173 | 26.2 | 2 | +5.1 | +2.78 |
| hypothetical proteins | comp19661_c0_seq2_No.1 | 29 | 5.3 | 1 | +2.35 | +7.35 |
| hypothetical proteins | comp20745_c1_seq11_No.1 | 117 | 7 | 3 | +1.67 | +3.17 |
| hypothetical proteins | comp11405_c0_seq1_No.1 | 230 | 42.6 | 5 | -1.9 | -2.91 |
| hypothetical proteins | comp4483_c0_seq2_No.1 | 168 | 23.1 | 5 | +17.86 | +2.86 |
